# Positive Autoregulation of an Acyl-Homoserine Lactone Quorum-Sensing Circuit Synchronizes the Population Response

**DOI:** 10.1128/mBio.01079-17

**Published:** 2017-07-25

**Authors:** Rebecca L. Scholz, E. Peter Greenberg

**Affiliations:** Department of Microbiology and Molecular and Cellular Biology Program, University of Washington, Seattle, Washington, USA; University of Minnesota Medical School

**Keywords:** bacterial communication, cell-cell signaling, transcriptional activation

## Abstract

Many proteobacteria utilize acyl-homoserine lactone quorum-sensing signals. At low population densities, cells produce a basal level of signal, and when sufficient signal has accumulated in the surrounding environment, it binds to its receptor, and quorum-sensing-dependent genes can be activated. A common characteristic of acyl-homoserine lactone quorum sensing is that signal production is positively autoregulated. We have examined the role of positive signal autoregulation in *Pseudomonas aeruginosa*. We compared population responses and individual cell responses in populations of wild-type *P. aeruginosa* to responses in a strain with the signal synthase gene controlled by an arabinose-inducible promoter so that signal was produced at a constant rate per cell regardless of cell population density. At a population level, responses of the wild type and the engineered strain were indistinguishable, but the responses of individual cells in a population of the wild type showed greater synchrony than the responses of the engineered strain. Although sufficient signal is required to activate expression of quorum-sensing-regulated genes, it is not sufficient for activation of certain genes, the late genes, and their expression is delayed until other conditions are met. We found that late gene responses were reduced in the engineered strain. We conclude that positive signal autoregulation is not a required element in acyl-homoserine lactone quorum sensing, but it functions to enhance synchrony of the responses of individuals in a population. Synchrony might be advantageous in some situations, whereas a less coordinated quorum-sensing response might allow bet hedging and be advantageous in other situations.

## IMPORTANCE

There are many quorum-sensing systems that involve a transcriptional activator, which responds to an acyl-homoserine lactone signal. In all of the examples studied, the gene coding for signal production is positively autoregulated by the signal, and it has even been described as essential for a quorum-sensing response. We have used the opportunistic pathogen *Pseudomonas aeruginosa* as a model to show that positive autoregulation is not required for a robust quorum-sensing response. We also show that positive autoregulation of signal production enhances the synchrony of the response. This information enhances our general understanding of the biological significance of how acyl-homoserine lactone quorum-sensing circuits are arranged.

## INTRODUCTION

Quorum sensing (QS) allows bacterial cells to monitor population density, relatedness, and diffusivity (1–6). QS systems have been shown to control cooperative bacterial behaviors, and virulence of a number of pathogens is attenuated by mutations in QS genes (7–13). We are interested in acyl-homoserine lactone (AHL)-mediated QS. The basic mechanism of AHL QS was first described for the luminescent marine bacterium *Vibrio fischeri* and was originally termed autoinduction (14). Autoinduction serves to activate the luminescence (*lux*) genes at sufficiently high *V. fischeri* densities. The autoinduction response requires two regulatory genes, *luxI*, which codes for an AHL synthase, and *luxR*, which codes for an AHL-dependent transcriptional activator (15). Each cell in the population constitutively produces basal levels of the AHL signal, which is freely permeable through cell membranes (16, 17). When the AHL reaches a sufficient concentration in the local environment, cells can activate expression of the *lux* genes, including *luxI* (18–20).

We have focused on related QS circuits in the pathogenic species *Pseudomonas aeruginosa*. This bacterium has two LuxI-LuxR-like genetic control circuits, LasI-LasR and RhlI-RhlR. Together these circuits activate hundreds of genes in *P. aeruginosa* (11, 21). Like the *V. fischeri* circuit, the *P. aeruginosa lasI* and *rhlI* genes are positively autoregulated by their cognate AHLs and LuxR homologs (22, 23). Positive autoregulation is a common characteristic of AHL-LuxR-type activator circuits (24). We are interested in exploring the costs and benefits of this QS-positive autoregulatory loop.

The original term for *V. fischeri* QS was autoinduction, and the autoinduction of luminescence was described prior to our understanding that *luxI* itself is positively autoregulated (14). Perhaps because of the similarity of the terms autoinduction and autoregulation, it is not uncommon to read that positive autoregulation of autoinducer synthesis is an essential element in QS (7, 25–27). We sought to use our model *P. aeruginosa* to test the essentiality hypothesis with LasR and LasI. We demonstrate that populations of *P. aeruginosa* engineered to produce the AHL signal at a steady rate regardless of cell population density show autoinduction responses similar to populations of cells with the wild-type (WT) positively autoregulated *lasI* gene. Analysis of individual cells in populations revealed that positive *lasI* autoregulation leads to more synchrony in the responses of individuals in the population.

## RESULTS

### *Pseudomonas aeruginosa* PAO-SC6 produces 3OC12-HSL constitutively in LB-MOPS with 0.5% l-arabinose.

**FIG 1:**
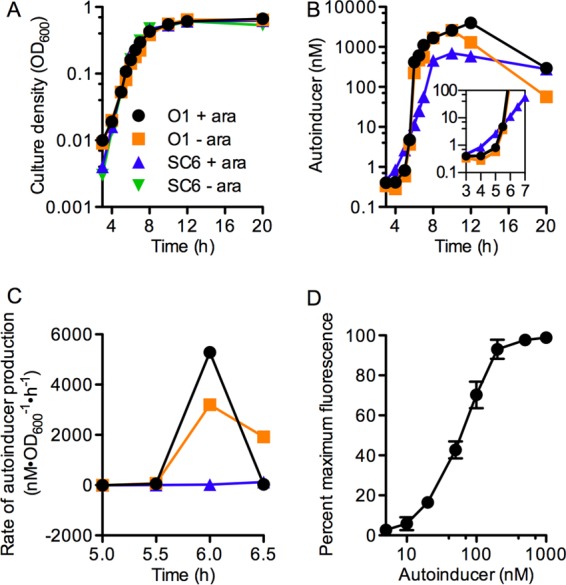
Production of 3OC12-HSL in *P. aeruginosa* PAO1 and PAO-SC6 and sensitivity of *P. aeruginosa* to 3OC12-HSL. (A) Growth curves of strains PAO1 and PAO-SC6. (B) 3OC12-HSL levels during growth (inset shows an expanded view of hours 3 to 7). (C) Calculated rates of 3OC12-HSL production in PAO1 and PAO-SC6 culture fluid. The symbol key in panel A shows strain PAO1 (O1) with or without 0.5% l-arabinose (ara), and PAO-SC6 (SC6) with (+) and without (−) l-arabinose for panels A to C. Strain PAO-SC6 without l-arabinose does not produce detectable levels of 3OC12-HSL and has been omitted from panels B and C. Cultures were grown in 75 ml of LB-MOPS broth with l-arabinose added as indicated. (D) Percent maximum fluorescence for the Δ*lasI* strain PAO-SC5 with pBS351 with 3OC12-HSL added at the indicated concentrations. Cells were grown in 160-ml medium without signal. At 3 h, 15-ml volumes were transferred to 125-ml baffled flasks containing 3OC12-HSL to give the desired final concentration of this autoinducer. Relative fluorescence units were measured at 6 h of growth. Error bars show the standard errors of the means (SEM) from three independent experiments.

The LasI-LasR circuit in *P. aeruginosa* produces and responds to the autoinducer 3-oxo-dodecanoyl-homoserine lactone (3OC12-HSL). In the WT strain PAO1, *lasI* is positively autoregulated. Strain PAO-SC6 has a deletion of the native *lasI* and an arabinose-inducible *lasI* inserted at the neutral *att* site on the chromosome. We first needed to measure 3OC12-HSL during growth of strain PAO-SC6 to determine whether it was produced at a constant level per cell. We also needed to determine whether cultures of PAO-SC6 and PAO1 reached a threshold autoinducer concentration at about the same time during growth. Growth of the two strains with or without l-arabinose was indistinguishable (Fig. 1A). Figure 1B shows concentrations of the autoinducer in culture fluid over the growth curve. There was a steep increase in autoinducer concentration in the WT cultures over a period between about 5 and 6 h followed by a plateau in autoinducer concentration as cells entered stationary phase. The WT autoinducer synthesis was not affected by l-arabinose. Strain PAO-SC6 did not make detectable levels of autoinducer in the absence of l-arabinose. In the presence of l-arabinose, the increase in autoinducer concentration paralleled the increase in cell mass, as expected if autoinducer synthesis per cell remained constant throughout growth. To further analyze the data shown in Fig. 1B, we calculated the rates of autoinducer synthesis over time between time points in the WT and the PAO-SC6 cultures [the difference in autoinducer concentration]/[the difference in cell density (OD_600_) × the difference in time between two time points] (Fig. 1C). In the WT PAO1, there was a sharp increase in the rate of synthesis between 5 and 6 h, and in strain PAO-SC6, the derived rate remained unchanged during logarithmic growth. These experiments confirm the positive autoregulation of signal production in the WT and the constitutive signal production in strain PAO-SC6.

For strain PAO-SC6, there is a small accumulation of autoinducer between 3 and 4 h of growth, whereas autoinducer concentrations in WT cultures remain low during this time period. Later in growth, the peak autoinducer concentration for the WT PAO1 is up to 10 times that of strain PAO-SC6 grown in the presence of l-arabinose (Fig. 1B). To probe the importance of these differences in autoinducer levels and to explore the sharpness of the QS threshold, we performed an autoinducer titration experiment. We used a QS-responsive reporter pBS351, which contains a *plasI-gfp* (*plasI* being the promoter for the *lasI* gene) transcriptional fusion, and measured the autoinducer concentration required to saturate the reporter response in a *lasI*-deficient strain (PAO-SC5). We chose the *lasI* promoter because *lasI* is among the earliest QS-dependent genes to respond to 3OC12-HSL (11). The minimum concentration at which we could detect a response was about 10 nM, and the response was saturated by about 100 to 200 nM 3OC12-HSL (Fig. 1D). The maximal signal concentration in PAO1 cultures reached about 10 times this saturating concentration; PAO1 makes significantly more autoinducer than necessary for a full response. This overproduction of autoinducer is a demonstrable cost to positive 3OC12-HSL autoregulation.

### Autoinduction in the absence of positive *lasI* autoregulation.

**FIG 2:**
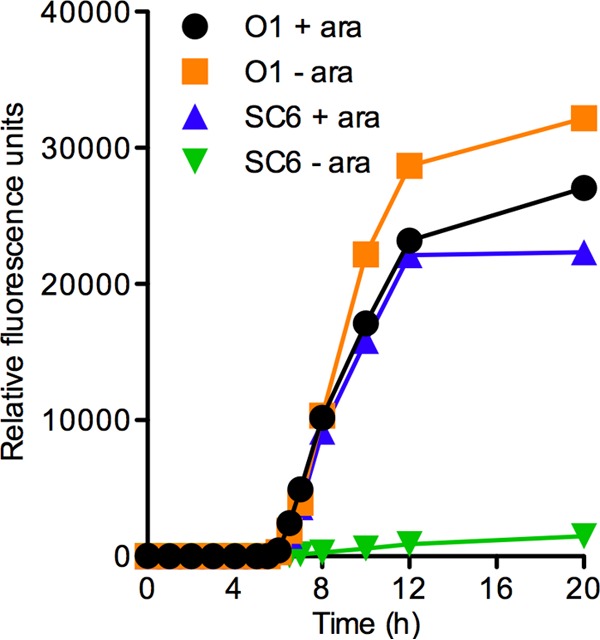
Positive 3OC12-HSL autoregulation is not necessary to induce a LasR-controlled promoter. Relative fluorescence units for strain PAO1 (O1) with or without 0.5% l-arabinose (ara) or strain PAO-SC6 (SC6) with or without ara. Both strains contained the *plasI-gfp* reporter. Cells were grown in 75 ml of LB-MOPS broth as described in Materials and Methods.

To further study the role of positive autoregulation of QS signal production, we monitored green fluorescence protein (GFP) fluorescence along the growth curve of PAO1 and PAO-SC6 populations carrying pBS351 and growing in LB-MOPS broth with or without l-arabinose (Fig. 2). There was a sharp increase in GFP fluorescence in the WT with or without l-arabinose and in strain PAO-SC6 with l-arabinose. The control, PAO-SC6 without l-arabinose, showed a very modest increase in fluorescence as population numbers increased over the growth curve. There may have been small differences in the responses of WT and PAO-SC6 in the first several hours of growth that were beyond the limits of our detection. Regardless, it is clear that autoinduction of *plasI-gfp* occurred in strain PAO-SC6 and that the response was quite similar to the response in the WT.

**FIG 3:**
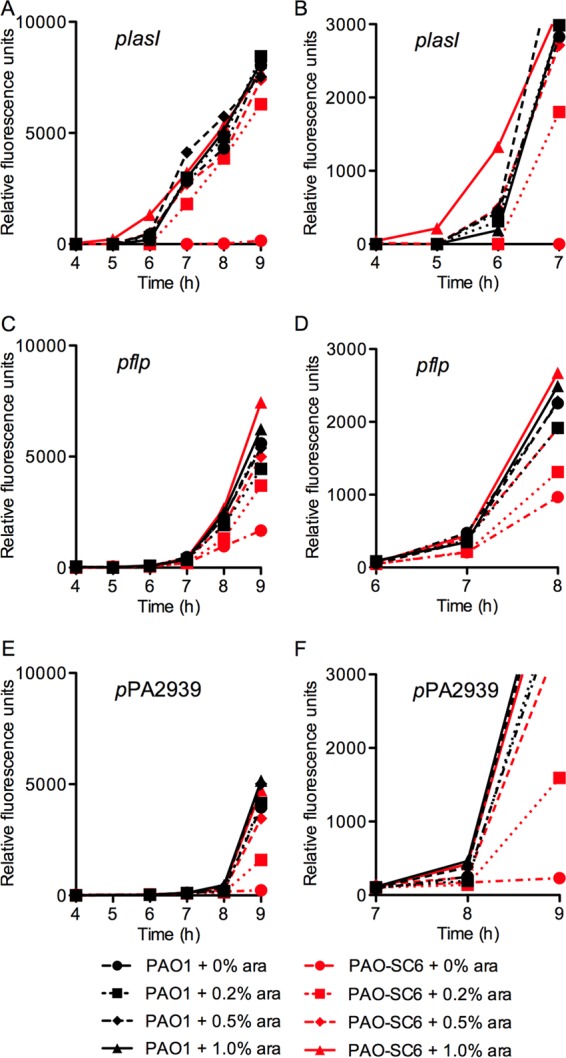
Multiple LasR-responsive promoters can be activated in the absence of positive 3OC12-HSL autoregulation. Activation of the promoters for *lasI* (A), *flp* (C), or PA2939 (E) in strains PAO1 and PAO-SC6. Panels B, D, and F show data from panels A, C, and E, respectively, in greater detail at the time of reporter induction. Cells were grown in 3-ml volumes as described in Materials and Methods.

We next wanted to test the effects of higher or lower basal rates of autoinducer production on activation of the *lasI* promoter. We did this by varying the concentration of l-arabinose in the growth medium (Fig. 3A and B). With 1% l-arabinose, autoinduction commenced somewhat earlier than it did with 0.5% l-arabinose, and at 0.2% l-arabinose, autoinduction was somewhat delayed. This shows that the basal activity of LasI affects the onset of autoinduction and that the signal is the trigger for activation of *lasI*.

### Autoinduction of late QS-controlled promoters in the absence of positive *lasI* autoregulation.

Sufficient *P. aeruginosa* LasR QS signal is required but not sufficient for activation of many genes in the LasR regulon (11, 28). The so-called late genes show an induction delay in the WT compared to early genes like *lasI*, presumably because their expression depends on additional regulatory inputs. We reasoned that late genes in particular should respond similarly in the WT and PAO-SC6 strains. Because of their delay in expression until late versus mid-logarithmic growth phase, sufficient autoinducer should have accumulated in the culture fluid of either bacterial strain to saturate LasR. Results with two late gene promoter-*gfp* fusions, *pflp-gfp* and *p*PA2939-*gfp* (*pflp* and *p*PA2939 being the promoters for the *flp* and PA2939 genes, respectively) are shown in Fig. 3C-F. Whereas *plasI-gfp* induction in the WT commences at about 5 to 6 h of growth, *pflp-gfp* induction commences between 6 and 7 h and *p*PA2939*-gfp* induction commences after 7 h. The first time point at which GFP was detected for either *flp* or PA2939 was not advanced by increasing the l-arabinose concentration from 0.5% to 1%, and it was not delayed by decreasing the l-arabinose concentration from 0.5% to 0.2%, although the intensity of GFP fluorescence does appear to depend on l-arabinose concentration for these later genes. In agreement with our previous publications (11, 28), autoinducer concentration is not the trigger for activation of these two genes.

### Positive autoregulation of signal production tightly synchronizes the responses of individual cells in a population.

**FIG 4:**
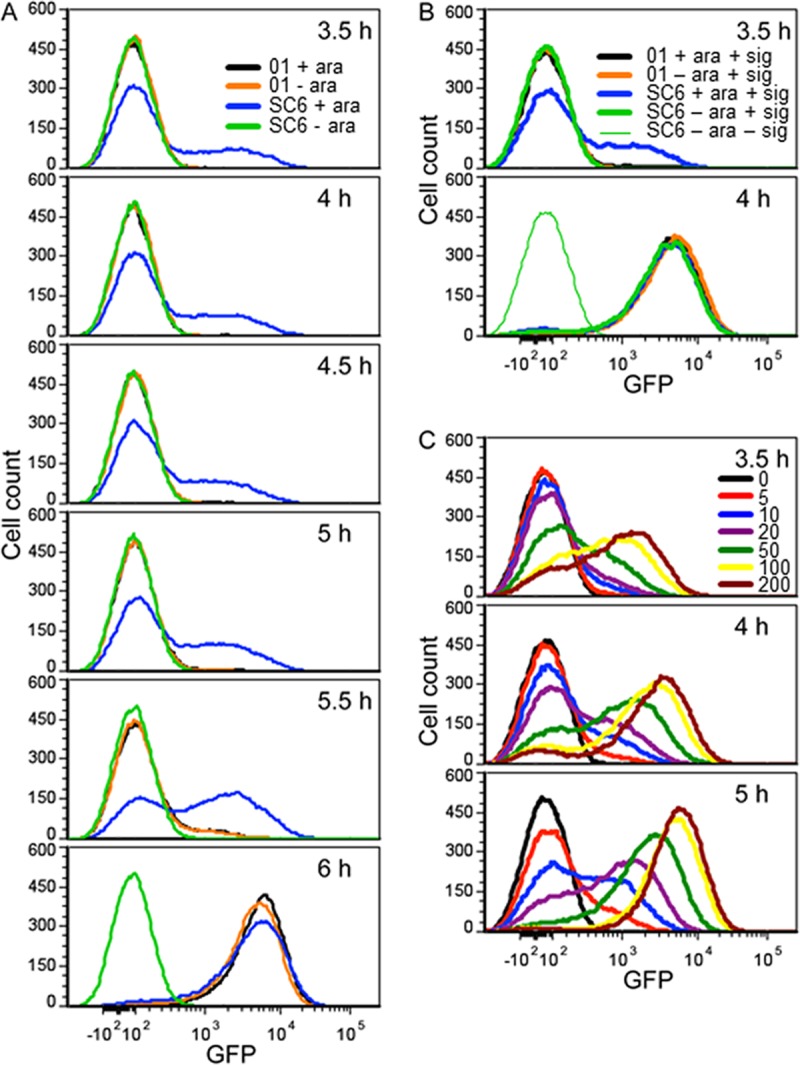
Positive 3OC12-HSL autoregulation synchronizes *plasI-gfp* expression. GFP expression in single cells was measured as described in Materials and Methods. All panels show cell counts with respect to GFP fluorescence. (A) Strains PAO1 (01) and PAO-SC6 (SC6) with the *plasI-gfp* reporter with or without 0.5% l-arabinose (ara). (B) As in panel A except 1 μM 3OC12-HSL (signal [sig]) was added at 3.5 h. PAO-SC6 without signal or l-arabinose was included as a negative control. (C) The *P. aeruginosa* Δ*lasI* mutant with the *plasI-gfp* reporter grown with added 3OC12-HSL (final concentrations are in nanomolar) at 3 h. Cells exposed to subsaturating levels of 3OC12-HSL show diminished synchrony of the response. Cells were grown in 15 ml of LB-MOPS broth as described in Materials and Methods.

To examine the autoinduction of *plasI-gfp* more closely, we monitored the responses of individual cells in populations by using flow cytometry (Fig. 4). For the WT PAO1, very few cells showed induced levels of GFP until about 5.5 h in culture when a small fraction of cells were expressing *plasI-gfp*. Thirty minutes later at 6 h, almost all of the cells were expressing *plasI-gfp*; the autoinduction response was tightly synchronized. In contrast, about 20% of PAO-SC6 cells showed partial GFP induction as early as 3.5 h in culture. The peak of induction occurred somewhere between 5.5 and 6 h, and at 6 h, the cell fluorescence levels of the WT and PAO-SC6 strains were similar (Fig. 4A). For a control, we also measured single-cell GFP induction in populations of the WT and PAO-SC6 to which we added 3OC12-HSL (final concentration, 1 μM) at 3.5 h postinoculation (Fig. 4B). In this experiment, both the WT and PAO-SC6 responses were tightly synchronized. Thus, the relaxed synchronization during autoinduction in PAO-SC6 cannot be attributed to factors unrelated to signal accumulation. We conclude that the autoinduction response in PAO-SC6 was less tightly synchronized than the response in the WT.

We note that there may be variations in *lasI* expression from the *araBAD* promoter (*paraBAD*) in strain PAO-SC6. However, AHLs can diffuse out of and into cells (16, 29). We do not expect the reduction in synchrony in strain PAO-SC6 to result from differences in *lasI* expression between cells. We also measured the response of the *plasI-gfp* reporter to subsaturating levels of 3OC12-HSL in the Δ*lasI* mutant PAO-SC5 and found that low levels of 3OC12-HSL early in growth also induce heterogeneous expression of this reporter within a population (Fig. 4C). Thus, the slow accumulation of 3OC12-HSL that we observed in PAO-SC6 populations (Fig. 1B) should be sufficient to produce the population heterogeneity we observed.

### Analysis of a late gene response in the WT and PAO-SC6 strains by flow cytometry.

**FIG 5:**
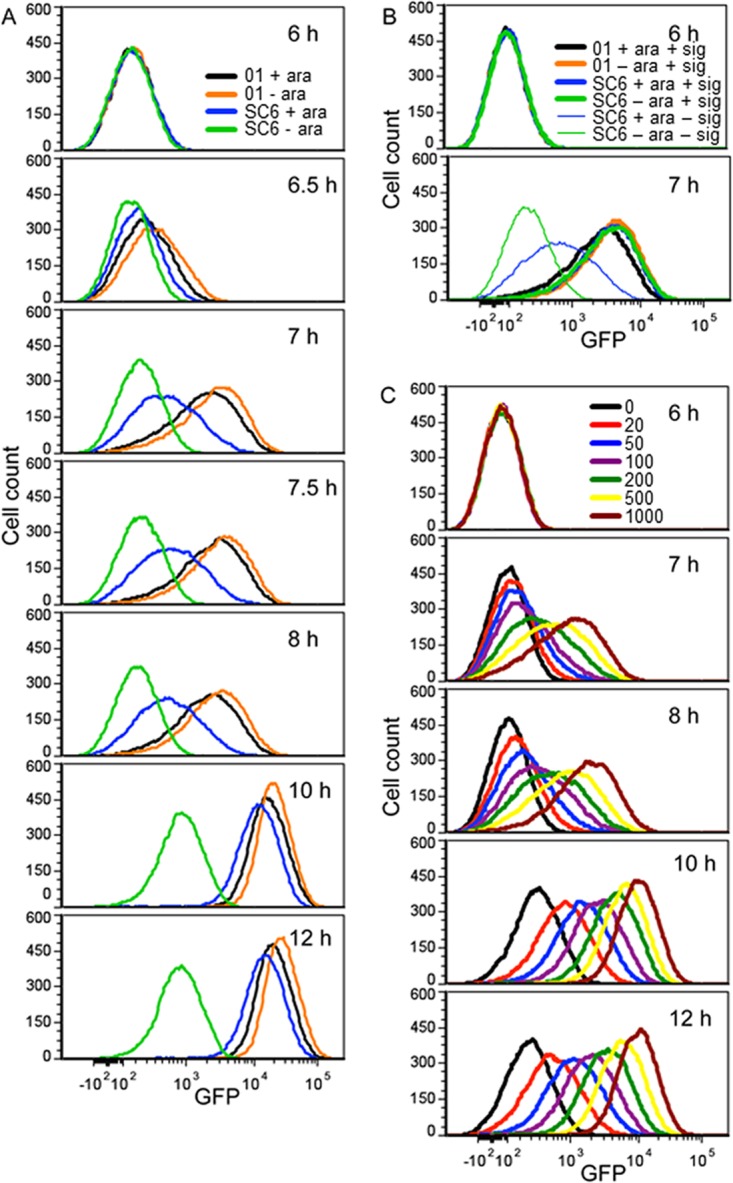
Positive *lasI* autoregulation facilitates maximal *p*PA2939*-gfp* expression. GFP expression in single cells was measured by flow cytometry. (A) Strains PAO1 (01) and PAO-SC6 (SC6) with the *p*PA2939*-gfp* reporter grown with or without 0.5% l-arabinose (ara). (B) When 1 μM 3OC12-HSL (signal [sig]) was added at 3.5 h, *gfp* expression was not advanced, but the levels of fluorescence of strains PAO1 and PAO-SC6 at 7 h were similar. Strain SC6 with ara and without sig or ara were included as controls. (C) 3OC12-HSL was added to cultures of the Δ*lasI* mutant PAO-SC5 with the *p*PA2939*-gfp* reporter at 3.5 h. The 3OC12-HSL signal was added at the indicated final concentrations in nanomolar. Cells were grown in 15 ml of LB-MOPS as described in Materials and Methods.

We hypothesized that late gene induction is tightly synchronized in both the WT and PAO-SC6 strains. This hypothesis is based on our findings that activation of late genes is delayed until after autoinducer concentrations in culture fluid have surpassed those required to saturate the *plasI-gfp* response (Fig. 1D and 3). To test this idea, we examined the responses of *p*PA2939*-gfp* in the WT and in PAO-SC6 by flow cytometry (Fig. 5). With the WT or PAO-SC6 strain, GFP was uninduced at 6 h; at 6.5 h, there was a small but detectable increase in fluorescence, and by 7 h, there was some autoinduction of the reporter in most cells. This comports with our hypothesis that autoinduction of a late gene will be tightly synchronized regardless of whether *lasI* is positively autoregulated or not; however, the situation is complicated. Although cells show autoinduction, *p*PA2939*-gfp* is not as strongly activated in PAO-SC6 cells as it is in WT cells at 7 and 7.5 h. By 10 h, the PAO-SC6 cells are almost as fluorescent as WT (Fig. 5A). For a control, we added 1 μM 3OC12-HSL to cultures after 3.5 h. This autoinducer concentration is roughly equivalent to that in WT cultures after 6 h of growth and exceeds that in 6-h PAO-SC6 cultures by about 2 log units (Fig. 1B). In this control experiment, GFP induction in PAO-SC6 cells is equivalent to that in WT (Fig. 5B). Thus, the increased time for PAO-SC6 cells to fully activate the *p*PA2939*-gfp* reporter can be attributed to the linear kinetics of autoinducer production by cells of this strain.

One simple explanation for the synchronized but slow induction of *p*PA2939*-gfp* in strain PAO-SC6 is that higher levels of autoinducer are required to achieve maximal induction; however, there are also other explanations. One way that we tested the idea that full activation of the PA2939 promoter requires high levels of autoinducer in comparison to activation of *lasI* was to add various amounts of 3OC12-HSL to populations of the *ΔlasI* mutant PAO-SC5 containing the *p*PA2939*-gfp* reporter plasmid and then monitor GFP fluorescence over time (Fig. 5C). In fact, maximum fluorescence per cell required high concentrations of autoinducer in the range of 500 to 1,000 nM.

## DISCUSSION

Positive autoregulation of autoinducer synthesis is a characteristic of AHL QS systems governed by LuxR family transcriptional activators, and it is common to many peptide signaling autoinduction systems in Gram-positive species (24, 30, 31). Positive signal autoregulation is not, however, a universal feature of all quorum-sensing systems. There are a few examples of AHL QS systems where the LuxR family, AHL-responsive transcription factor is a repressor rather than an activator, and in these systems, the cognate *luxI* homolog is not positively autoregulated (32–35). For example, in *Pantoea stewartii*, the LuxR homolog EsaR binds to target DNA in the absence of its cognate AHL and blocks transcription. At sufficient AHL concentrations, EsaR is released from the target DNA (33). Because of the commonality of positive autoregulation of signal synthesis by LuxR-type transcriptional activators, and perhaps because the terms autoinduction and autoregulation can be confused, positive autoregulation has been described as an essential part of autoinduction by these transcriptional activators. Our experiments with *P. aeruginosa* demonstrate that this is not the case (Fig. 1 and 2). We hope that this work will help to disentangle autoinduction from autoregulation and also contribute to our understanding of how QS signaling information is “encoded” in positively autoregulated systems (36). Perhaps measurements made in this work will also help to hone future models of QS signaling networks.

If positive autoregulation of AHL signal synthesis is not a required element of the autoinduction response, then why is it a common characteristic of the LuxR-activated circuits? At the population level, positive autoregulation of signal production would be expected to result in a threshold QS response versus a graded response for constitutive signal production. This has been shown computationally and in the *lux* QS circuit of *V. fischeri* (37). We observed similar threshold versus graded responses when measuring the accumulation of autoinducer in strains PAO1 and PAO-SC6, respectively (Fig. 1B). Interestingly, we do not observe a clear difference in population-level induction of *plasI-gfp* expression between strains PAO1 and PAO-SC6 (Fig. 2). This difference from that observed previously in *V. fischeri* (37) could be due to many factors, not least of which are the growth and measurement conditions used. We measured GFP expression in logarithmic phase cultures, as opposed to periodically-diluted steady-state cultures at specific cell densities (37). The rapid accumulation of both cells and autoinducer in our experiments may have masked the differences observed in experiments with steady-state cultures. Additionally, the stability of the GFP used (stable version versus the short-half-life version) or differences in the affinity of the LuxR homolog to its respective promoter-*gfp* reporter could contribute to the differences between our population-level fluorescence measurements and those of Haseltine and Arnold (37). At the single-cell level, however, we too find that the positively autoregulated QS system produces a more synchronized threshold-like activation pattern compared to the more graded activation pattern observed in bacterial groups without positive signal autoregulation (Fig. 4).

We uncovered two consequences of positive signal autoregulation when we examined the responses of individual *P. aeruginosa* cells in populations undergoing autoinduction of LasR-dependent-promoter-controlled *gfp* reporters. First, for a so-called early QS-responsive gene (activation of early genes can be advanced by inclusion of autoinducer in the growth medium; the autoinducer is the trigger for gene activation), *lasI*, positive autoregulation enhanced synchronization of the response (Fig. 4). Second, so-called late genes, for which autoinducer is required but not sufficient for activation (the autoinducer is not the trigger for activation) are synchronized tightly, but cells show a relatively slow response in the absence of positive autoregulation (Fig. 3 and 5). Improved synchronization seems intuitive: when signal is positively autoregulated, the length of time that the population is exposed to intermediate autoinducer concentrations (above the threshold for activation but below the saturating level) is short. Intrinsic heterogeneity among cells would be overridden by the quickly saturating signal concentrations. Delaying or preventing saturation of a signaling system by removing one or more regulatory inputs has been shown to also increase heterogeneity in *V. harveyi* QS, indicating that rapid saturation of signaling systems may be a general mechanism to synchronize QS populations (38). We did not anticipate the late gene response, as signal levels are quite high by the time late gene expression commences. We do not know the mechanistic basis for this slow cellular increase in late gene expression, but there are several possible explanations. For example, it may have to do with LasR-binding affinities and competition with early QS-dependent genes for LasR.

We note that positive autoregulatory loops are not unique to QS systems. Experimentation and modeling show that such autoregulatory loops can reduce noise, create a bistable state, and decrease cost (39–43). With AHL-responsive LuxR family transcriptional activators, positive autoregulation of signal production can affect stability in populations. There should be hysteresis with the cell density required to achieve a threshold level of autoinducer being much higher than the density required to deactivate the system. We have not addressed this possibility experimentally.

It is of interest to determine whether responses of quorum-sensing-dependent genes governed by repressors like that of *Pantoea stewartii* for example (33) show tight synchrony in their response. We also note that the autoinducer-2 activation of *Vibrio harveyi* luminescence, which does not involve a LuxR homolog, shows bistability (44). Bistability is thought of as a bet-hedging strategy that can facilitate success in an environment where conditions are variable. A more tightly synchronized response, which is perhaps further committed by positive autoregulation of autoinducer synthesis might be particularly well suited to situations where uninduced individuals are at risk from extrinsic or intrinsic factors or where cooperation is essential for success.

## MATERIALS AND METHODS

### Bacterial strains, plasmids, and culture conditions.

The *Pseudomonas aeruginosa* and *Escherichia coli* strains used are listed in Table 1. Bacteria were grown in Luria-Bertani (LB) broth (10 g tryptone, 5 g yeast extract, 5 g NaCl per liter) with 50 mM 3-(*N*-morpholino)propanesulfonic acid (MOPS) (pH 7.0) (LB-MOPS broth) or on LB-MOPS agar (LB-MOPS broth plus 1.5% agar) and supplemented as noted. Antibiotics were used for plasmid maintenance of selection at the following concentrations as appropriate: for *E. coli*, 100 μg/ml ampicillin (Ap), 10 μg/ml gentamicin (Gm), and 10 μg/ml tetracycline (Tc); for *P. aeruginosa*, 100 μg/ml Gm, 100 μg/ml Tc, 150 μg/ml carbenicillin (Cb). Where indicated l-arabinose (final concentration of 0.5% unless otherwise indicated) or the QS signal 3OC12-HSL at the indicated concentration was added to the growth medium. The QS signal was dissolved in ethyl acetate, and the solution was dried on the bottom of the culture vessel prior to the addition of cultures. Bacteria were grown in 3-ml volumes in 18-mm tubes, 15-ml volumes in 125-ml baffled flasks, or 75-ml volumes in 500-ml baffled flasks as noted and grown at 37°C with shaking (250 rpm). Starter cultures of *P. aeruginosa* were from single colonies obtained by streaking from a frozen stock.

**TABLE 1:** Bacterial strains and plasmids used in this study

Strain or plasmid	Description or relevant genotype	Reference or source
Strains		
*P. aeruginosa* PAO1	Wild-type prototroph	56
*P. aeruginosa* PAO-SC5	*lasI* deletion mutant of PAO1	This study
*P. aeruginosa* PAO-SC6	PAO1 Δ*lasI paraBAD*-*lasI*	This study
*E. coli* DH5α	*E. coli* cloning vehicle	Invitrogen
*E. coli* S17-1	*thi pro hsdR recA* RP4-2 (Tet::Mu) (Km::Tn*7*)	57
Plasmids		
pJN105L	*paraBAD*-*lasR*; Gm^r^	54
pSC11	*plasI*-*lacZ* reporter; Ap^r^	55
pEXG2-Δ*lasI*	Gene replacement vector; contains *lasI* flanking regions to delete codons 31 to 191 of *lasI*; *sacB* Gm^r^	46
pSW196	Mini-CTX2 with *paraBAD* promoter; Tet^r^	47
pSW196-RBS-*lasI*	*attB* integration plasmid with arabinose-inducible *lasI*; Tet^r^	This study
pFLP2	Expresses Flp recombinase; *sacB* Ap^r^ Cb^r^	49
pPROBE-GT	Promoterless *gfp*; Gm^r^	51
pBS351	*plasI-gfp*: pPROBE-GT with *lasI* promoter (*plasI*); Gm^r^, −282 to +223^a^	58
pBS383	*pflp-gfp*: pPROBE-GT with *flp* promoter (*pflp*); Gm^r^, −283 to +100^a^	This study
pBS347	*p*PA2939-*gfp*: pPROBE-GT with PA2939 promoter (*p*PA2939); Gm^r^, −290 to +223^a^	This study

a The numbers indicate the coordinates of the *P. aeruginosa* gene fragments relative to the translational start site of the relevant gene.

### Strain and plasmid construction.

We used Qiagen kits for routine DNA purification. QIAprep spin miniprep kits were used for plasmid preparation, and QIAquick PCR purification kits and QIAquick gel extraction kits were used for PCR product purification and gel extraction, respectively. A chromosomal deletion of *lasI* (PAO-SC5) was constructed by introducing pEXG2-Δ*lasI*, which includes DNA homologous to the flanking regions of *lasI*, into strain PAO1 by standard protocols (45, 46). To construct a strain with a chromosomal copy of an arabinose-inducible *lasI* gene, we first assembled pSW196-RBS-*lasI*. To make this plasmid, a PCR fragment with engineered NotI sites flanking bp −20 to +612 relative to the *lasI* translational start was ligated to NotI-digested pSW196 (47) following the *araBAD* promoter. This plasmid was built on a mini-CTX2 backbone, which was designed to integrate into the *attB* site of *P. aeruginosa* (47, 48). To construct a *P. aeruginosa* mutant with a single copy of *lasI* controlled by the arabinose promoter, we mated *E. coli* strain S17-1 carrying pSW196-RBS-*lasI* with the Δ*lasI P. aeruginosa* strain PAO-SC5. We used antibiotic selection to obtain an isolate with pSW196-RBS-*lasI* in the *attB* site. We then introduced pFLP2 and used Flp recombination to remove the antibiotic resistance cassette from the integrated plasmid (49). Finally, we used sucrose to counterselect *sacB* and cure the strain of pFLP2 (50).

To construct the pBS series of LasR-inducible promoter reporter plasmids described in Table 1, we cloned PCR-generated DNA fragments with SalI and BamHI overhangs into SalI-BamHI-digested pPROBE-GT (51) by using standard procedures. All plasmid and mutant constructs were confirmed by DNA sequencing.

### Growth curves and population fluorescence measurements.

Starter cultures for growth curves were grown from a single colony inoculated into 3 ml of LB-MOPS broth. When cultures reached an optical density at 600 nm (OD_600_) of between 0.05 and 0.2, they were used to start experiments at an initial OD_600_ of 0.001. Optical densities were measured by using a Genesys spectrophotometer with a 1-cm-path-length cuvette. Either 3OC12-HSL or l-arabinose or both were included as noted. Relative fluorescence units (RFUs) (485-nm excitation, 535-nm emission) and OD_600_ in 20-μl samples in black-walled transparent-bottom 384-well plates were measured by using a BioTek synergy H1 microplate reader.

### Flow cytometry.

We used flow cytometry to measure individual cell responses in populations of *P. aeruginosa*. Cells were grown in 15 ml of LB-MOPS broth. The cultures were sampled at the times indicated, and the cells were pelleted by centrifugation, washed twice in phosphate-buffered saline (PBS) (pH 7.4), suspended in 2.5% paraformaldehyde in PBS, and fixed at 37°C with shaking for 10 min. The fixed cells were washed three times in PBS and then stored in PBS at 4°C. Cells were analyzed by using a BD LSR II flow cytometer and BD FACSDiva software. Cells were gated to exclude debris based on forward scatter (FSC) and side scatter (SSC). Twenty thousand events were acquired for each sample. GFP was excited with the blue laser (488 nm), and the emitted light was collected via a 530/30 nm filter. Data were analyzed using FCS Express 4 software.

### Autoinducer bioassays.

We used a bioassay to measure 3OC12-HSL as described elsewhere (52, 53). Briefly, we extracted the QS signal 3OC12-HSL from culture fluid in ethyl acetate acidified with 0.01% glacial acetic acid and concentrated the autoinducer 10-fold. We used an *E. coli* bioassay strain containing *lasR* and a LasR-inducible promoter fused to *lacZ* (54, 55) to measure 3OC12-HSL, and we prepared a standard curve by using synthetic 3OC12-HSL (RTI International) to calculate signal concentrations in cultures as described elsewhere (53).
